# Machine Learning Methods to Predict the Academic Performance of Pre‐Clinical Dental Students Based on Pre‐University Information

**DOI:** 10.1155/ijod/5468404

**Published:** 2026-06-11

**Authors:** Merve Koseoglu, Remya Ampadi Ramachandran, Merve Botsalı, Cortino Sukotjo

**Affiliations:** ^1^ Department of Prosthodontics, Faculty of Dentistry, University of Sakarya, Sakarya, Türkiye; ^2^ 1DATA Consortium, Computational Comparative Medicine, Department of Mathematics, Kansas State University, Olathe, Kansas, USA, k-state.edu; ^3^ Department of Prosthodontics, University of Pittsburgh, School of Dental Medicine, Pittsburgh, Pennsylvania, USA, pitt.edu

## Abstract

**Background:**

Information regarding the prediction of dentistry students’ academic performances using machine learning (ML) techniques is lacking. The objective of this study was to predict the academic performance of pre‐clinical dentistry students using ML techniques based on their pre‐university information.

**Methods:**

A total of 96 data points, including graduation years, genders, high school grade point average (GPA), step 1 university exam score (Step 1 UES), Step 2 UES, total UES, university exam ratings (UER), high school types (HSTypes), hometowns, parent educations (PEs), and parent incomes (PIs) from dentistry grads, were collected. Various ML regression techniques were validated to predict the academic performance of dentistry students in this cross‐sectional study.

**Results:**

Preliminary findings from the dataset indicated that UESs or rankings were more predictive than other demographic features, such as HSType, gender, graduation year, and hometown. The selection of target variables has resulted in two major studies. The initial study employed overall preclinical scores as the target variable, whereas the subsequent study utilized letter grades from specific preclinical courses, including dental morphology manipulation, dental materials I, prosthodontics I, and prosthodontics II, for ML models. The performance measures of these models demonstrated the capability of ML models to evaluate student performance in relation to demographics, high school performance, and admission scores. Regression models exhibiting an *R* ^2^ value of 0.74 or greater, alongside a classification accuracy beyond 70% for a balanced dataset of preclinical subject scores, demonstrated that ML models can predict student achievement in both overall and subject‐specific grades.

**Conclusion:**

The present study revealed the utilization of ML techniques to predict the academic performance of dentistry students successfully.

## 1. Introduction

Data are the vital asset for educational organizations, as it provides valuable insights, knowledge, and intelligence for authorities. In colleges and universities, students constitute the principal source of data generation. They are fundamental entities within the academic environment, where data is derived from quizzes, assignments, and midterm and final examinations. Various elements influence student performance, including personal, social, historical, and academic background, familial influences, social media interactions, and organizational factors, which serve as force multipliers in their learning behavior and ultimately impact educational outcomes [1, 2]. Predicting students’ academic achievement is receiving significant interest in education [3, 4].

The annual selection of students in undergraduate programs is conducted under the central administration of the Turkish university entrance exam, which has two steps in Turkey [5]. The first step is the basic proficiency test, while the second step is a more comprehensive exam [6]. Those who passed the university entrance exam can start studying dentistry. In Turkey, a 5‐year dentistry program integrates academic and practical training. Each dentistry faculty has its own curriculum and grading system. The first‐year curriculum at the Sakarya University Faculty of Dentistry includes lectures, laboratory work, and preclinical exercises in basic sciences, dental morphology and manipulation, and dental materials. During the second and third years, students are expected to enhance their preclinical skills, comprehend the application of fundamental scientific ideas in clinical contexts, and engage in clinical applications. Students take pre‐clinical lessons during the first, second, and third years. During the fourth and fifth years, students engage in primary clinical treatments alongside courses that enhance their technical and diagnostic abilities [7].

Machine learning (ML) is a widespread technology that predicts data across various domains. Academicians and administrators use data to forecast a student’s performance at admission, predict job prospects upon course completion, assess dropout rates based on aggregate statistics from the entire student cohort, or evaluate an individual student’s likelihood of success or failure in subsequent grades [8–12]. The substantial increase in research contribution within big data and ML has similarly promoted the advancement of learning analytics and learning in education [8].

Diagnostic and predictive modeling in dentistry has progressed from traditional statistical methods to more sophisticated ML and deep learning (DL) models. Compared to traditional statistical models, the introduction of ML algorithms improved the detection and accuracy of these predictions. DL, the subset of ML models, particularly convolutional neural networks (CNNs), revolutionized dental imaging by automatically extracting features from radiographs and 3D scans. The challenges of interpretability, data quality, and technical complexity include the need for expert‐driven preprocessing and tuning across ML and DL. Automated ML has emerged as a promising solution that makes advanced analytics more accessible and scalable for clinical use, potentially improving diagnostic accuracy and workflow integration [13]. Nowadays, generative AI models are rapidly advancing the biomedical field and its data analysis, simulation, and interpretation tasks. Its capability to generate realistic synthetic data, uncover latent patterns, and automate complex analytical operations is not only enhancing current research capabilities but also redefining the future of precision medicine [14]. ML models are also used to analyze demographic data and related social factors for predicting student performance in both clinical and academic components of their college education [15]. The literature review showcases the application of various ML models in different dentistry applications, including periodontics, orthodontics, restorative dentistry and prosthodontics, oral medicine and pathology, maxillofacial surgery, and endodontics. Independent of the field of application, the key aspect of every study is the appropriate selection of ML models based on the dataset for the study [16].

Fundamental data science principles and best practices, along with the studies reviewed so far, confirm that ML model selection depends on the target data and the specific objectives of the study. For example, CNN models are often employed where we deal primarily with images or spatially or sequentially correlated data types. Recurrent neural networks (RNNs) and long short‐term memory (LSTM) will be effective with sequential data such as speech, time series, and natural language. Tree‐based models, including random forest (RF), support vector machine (SVM), linear regression (LR), and logistic regression, are primarily used for classification and regression tasks involving tabular datasets [17]. Similarly, the selection of ML models in our proposed study involves the selection of two distinct approaches in ML model selection: one for classification‐based predictions and the other for regression‐based prediction of academic performance, along with models to assess the temporal progression of student performance across academic years to capture sequential dependencies. For example, in the present study, different ML models, including LR, LSTM regression with ReduceLROnPlateau, GBR, and extreme gradient boosting (XGB), were implemented due to their improved performance and accuracy for the given dataset.

Several studies investigated the prediction of university students’ performances using different prediction models in other fields [2, 4, 18, 19] and in dentistry to predict dental students’ academic performance, and the classification accuracy of the proposed models ranged from 29% to 57%, ranked from highest to lowest as follows: RF, SVM, DT, and LR [17]. However, it is well known that the education systems of each country and institution are different, which may affect the success of ML techniques. Entrance tests will undoubtedly be one of the most contributing predictive factors, since they are used to ascertain student enrollment [20]. In this study, we are also exploring the features’ contribution towards preclinical score assessment and predictions. The present study aimed to explore the feasibility of predicting the academic performance of the pre‐clinical dentistry students using their pre‐university data, including their gender, high school type (HSType), hometown city, parent education (PE), parents’ monthly income, their university entry exam results, ratings, and graduation years, and to evaluate the value of using ML in admissions and students’ assessment. The present study hypothesized that ML models would exhibit good predictive performance in predicting preclinical dental students’ academic performances using their data.

## 2. Materials and Methods

### 2.1. Data Collection

The study protocol was approved by the Sakarya University Faculty of Medicine research ethics committee (protocol number: E–43012747‐050.04‐428167–162) and conducted in accordance with the 1964 Declaration of Helsinki. Data were obtained from 2023 and 2024 graduates of the Faculty of Dentistry at the Sakarya University, Turkey. The criteria for inclusion and exclusion of data in the study were as follows: Inclusion criteria: all dental graduates have comprehensive records. Exclusion criteria: incomplete academic records, including students who have not completed all courses, such as those who transferred to other universities without graduating from the Sakarya University Faculty of Dentistry. Participants were informed about the study’s purpose, and written consent forms were obtained from all participants. A total of 96 students graduated during this period, and 100% of the eligible cohort was included in the study, as all graduates possessed comprehensive academic records meeting the inclusion criteria. No participants were excluded due to incomplete records or institutional transfers, ensuring a complete census and eliminating potential selection bias. Individual coding was used to protect each student’s identity as data points from dentistry graduates were gathered [17].

Graduation year [18], gender [2], high school grade point average (GPA) [2], step 1 university exam score (Step 1 UES) [6], Step 2 UES [6], total UES [6], university exam ratings (UER) [6], HSType (HSType) [6], hometown [2], PE [2], and parent income (PI) [2] as the input variables or features for the ML models (Table 1). The grades of dentistry students from all courses taken from the beginning of the 1^st^ year to the end of the 5^th^ year were recorded. The examination grading method was divided into six categorical classes based on percentage scores: AA (90–100, very good success), BA (85–89.99, good–very good success), BB (75–84.99, good success), CB (70‐74.99, moderate–good success), CC (60‐69.99, moderate success) and FF (0‐59.99, fail) [7].

**Table 1:** Inputs’ Descriptions.

Input	Description with example
Graduation year	Graduation year of the student, for example, 2023 or 2024
Gender	Gender of the student, for example, male and female
High school GPA	GPAs of the studied high school
Step 1 UES	The score the student received on the 1^st^ step of the university entrance exam
Step 2 UES	The score the student received on the 2^nd^ step of the university entrance exam
Total UES	The total UES score is determined by adding 40% of the Step 1 UES, 60% of the Step 2 UES score, and the high school success score, which is mainly derived from high school GPA
UER	The student’s ranking among other students based on the score he/she received in the university entrance exam
HSType	The type of high school the student graduated from, for example, public or private
Hometown	The hometown where the student comes from, for example, metropolis or small city
PE	The education of the students’ parents, for example, primary school, secondary school, high school, university, postgraduate degree
PI	Monthly income of students’ parents in Turkish lira.

### 2.2. ML Methods

#### 2.2.1. Feature Importance Assessment

In this study, two different approaches were considered to measure the features that can contribute more to preclinical score prediction. In the first approach, SelectKBest with f_regression ranks the features in the dataset by their importance with respect to the continuous target variable. It is mainly employed in LR problems and ranks features based on the *F*‐statistic or *p*‐value calculated, then performs top “k” feature selection with the highest *F*‐statistic or lowest *p*‐value. The second approach used feature_importances_ attribute of RandomForestRegressor object, which indicates how much each feature contributes to the ML model’s predictions [21]. Both feature selection and model‐based importance selection were conducted totally on the training dataset, ensuring that the test dataset remained completely independent for model evaluation.

#### 2.2.2. Student Performance Prediction Models

Overall, student preclinical performance score prediction incorporated two prediction schemes, where the first approach considered university admission scores and student demographics as input variables to predict the average 3‐year preclinical score. In the second approach, year‐wise performance scores have been predicted based on the learning knowledge from the university admission scores and student demographics as the input variables, as well as based on the trend based on the scores obtained for the first, second, and third years of their study. This study implements another ML model that utilizes individual preclinical subject scores as the target variable.

##### 2.2.2.1. LR

LR is a fundamental statistical and ML technique used to model the relationship between one or more input features and a continuous target variable. The input features can be both numerical and categorical. The model assumes a linear relationship between the input or independent variables and the dependent or target variable, estimating coefficients that minimize the difference between the predicted and actual values. This method is widely used for its simplicity, interpretability, and efficiency in predicting target variables that are continuous in nature based on the historical data [21].

##### 2.2.2.2. LSTM Regression with ReduceLROnPlateau

LSTM networks are a type of RNN designed mainly to handle sequential data, making them effective for continuous target prediction when time‐dependent patterns exist in the data. LSTMs capture long‐term dependencies by maintaining memory cells that control information flow through input, forget, and output gates. To optimize training, ReduceLROnPlateau is often employed as a learning rate scheduler, which reduces the learning rate when model performance plateaus, helping avoid overfitting and improving convergence [22].

##### 2.2.2.3. Gradient Boosting Regression

Gradient boosting regression is an ensemble learning technique that builds an efficient predictive model by sequentially training decision trees, where each tree corrects the errors of its predecessors. GBR minimizes a loss function using gradient descent, making it highly effective for capturing complex, non‐linear relationships [21, 23].

##### 2.2.2.4. XGB

XGB regression models are a highly optimized, efficient, scalable, and portable implementation of GBR algorithms. With the support of built‐in regularizations, a more robust model that is less prone to overfitting issues has been generated [23–25].

### 2.3. Overall ML Modeling Strategy

In the data preprocessing step, categorical predictors such as gender, HSType, Hometown, and PE were already represented as numeric category codes in the dataset and were therefore used directly as model inputs. Continuous predictors, such as High school GPA, Step 1 UES, Step 2 UES, total UES, UER, and PI, were retained in their original numerical form, and feature scaling using StandardScaler was applied only to models sensitive to feature scaling, such as LR and DL models. On the other hand, tree‐based models such as gradient Boosting and XGBoost were trained using unscaled predictors, as scaling does not affect split‐based ensemble methods. For the subject‐wise preclinical performance multiclass classification models, LabelEncoder was applied only to the target variables to convert letter grade labels into numerical form for model training [21].

In this study, we implemented two complementary evaluation methods to balance statistical reliability with interpretability, given the relatively small sample size (*N* = 96). First, the model generalization performance was estimated using stratified repeated resampling through five‐fold cross‐validation (CV) (shuffle = True, random_state = 42) and reported the mean ± standard deviation of *R* ^2^, mean absolute error (MAE), and root mean‐squared error (RMSE) across folds. Preprocessing with feature scaling was fitted on the training portion of the data and subsequently applied to the corresponding held‐out fold, which helped to control the data leakage issues. LR used standardized predictors, whereas tree‐based models, such as gradient boosting regression and XGB were trained on unscaled predictors, as scaling does not influence split‐based ensemble models. Second, to generate interpretable visualizations of predicted and observed year‐wise academic trajectories, models were additionally trained using a single 70:30 train–test split (random_state = 42) and plotted on the held‐out test dataset. The input variables were organized into two categories such as static features and sequential features. Static features included admission and demographic variables (graduation year, gender, high school GPA, Step 1 UES, Step 2 UES, total UES, UER, HSType, hometown, PE, and PI), which remain constant for each student. Sequential features consisted of previous year academic performance scores representing temporal progression across preclinical training (P_Y1 and P_Y2). The prediction targets included Year 3 performance (P_Y3) and the overall 3‐year average performance (P_T). This approach enabled the implementation of a temporal DL approach, with a specific LSTM branch to process sequential input features and a dense branch to handle static input features. The outputs of the two branches were concatenated before the final regression layer to produce the predicted performance outcome. This architecture helps the model learn both static predictors and sequential academic progression across years. Significantly, predictions for Year 3 performance (P_Y3) and the overall average performance (P_T) were generated using only information available prior to Year 3, thereby avoiding target leakage. The LSTM network trained using MSE loss with early stopping and adaptive learning rate reduction (ReduceLROnPlateau) helped to improve training stability and mitigate overfitting.

In addition to regression‐based prediction of academic performance, classification analysis was conducted to predict subject‐level grade outcomes. XGB was used as the primary classification model due to its ability to capture nonlinear relationships between predictors and grade categories. Because the grade distributions across subjects were imbalanced, a RandomOverSampler strategy was applied to the training data to improve the representation of minority classes. Oversampling was performed only on the training subset after the train–test split, while the test dataset retained the original class distribution to ensure unbiased model evaluation. Model performance was assessed using precision, recall, F1‐score, and confusion matrices, with support values reported for each grade category.

Additional measures, such as hyperparameter tuning and CV techniques, were incorporated with the ML models to avoid overfitting problems. In LSTM models, Dropout layers were included to improve generalization and prevent overfitting. While in GBR models, hyperparameters like learning rate, tree depth, and the number of estimators were carefully tuned to balance accuracy and overfitting. In XGB models, apart from the hyperparameter tuning, the model’s in‐built regularization functions L1 (Lasso) and L2 (Ridge), tree constraints to control the model’s complexity, help reduce variance and prevent overfitting issues.

The performance of the ML regression models is typically evaluated using metrics such as the *R* ^2^ score (coefficient of determination), MSE, RMSE, and MAE to quantify prediction accuracy. The *R* ^2^ score measures how well the independent or input variables explain the variability of the target or dependent variable, with values closer to 1 indicating a better fit. MSE and RMSE quantify the average squared difference between the actual and predicted values. MAE calculates the average absolute differences, making it less sensitive to outliers compared to MSE. The models predicting student performance in individual preclinical subjects, classified as classification models, were assessed using performance metrics such as accuracy and F1 scores.

All model development steps, including feature selection, preprocessing, oversampling for subject‐wise classification assessment, and model training, were performed exclusively on the training dataset within each validation procedure. The test dataset contained the original data distribution and was solely used for the final model performance evaluation and visualization. This data leakage‐safe workflow ensures that the reported performance metrics reflect true model generalization performance instead of unintended information leakage between training and evaluation data.

## 3. Results

Prediction models for the preclinical academic performance assessment considered graduation year, gender, high school GPA, Step 1 UES, Step 2 UES, total UES, UER, HSType, hometown, PE, and PI as the input variables or features for the ML models. Mainly, two separate studies have been devised based on the target variable selection. The first study considered the overall preclinical score and preclinical score for each year as the target variable, while in the second study, the letter grade of individual preclinical courses, such as dental morphology manipulation, dental materials I, prosthodontics I, and prosthodontics II, was taken as the target variable of the ML models.

Analysis was performed on the full cohort of 96 graduates (100% participation). The dataset reflected a balanced distribution across primary predictors; the mean UES for step 1 and step 2 were 413.6 ± 7.4 and 429.8 ± 6.6, respectively. Demographic variables, including gender, HSType, and parental income, were distributed representatively across the cohort, providing a robust and comprehensive baseline for the subsequent ML models without any missing observations.

### 3.1. Validation Workflow and Hyperparameter Tuning

The model performance for predicting overall academic performance was evaluated using an 80:20 train–test split (random_state = 42). StandardScaler was used to normalize the predictor variables. The scaling parameters were fitted on the training data and then used on the test data to prevent data leakage. *R* ^2^ and MAE were mainly used to evaluate the model performance on the held‐out test set. To evaluate the model strength with the limited sample size (*N* = 96), repeated 80:20 hold‐out resampling was conducted using various random seeds, and performance metrics are presented as mean ± standard deviation across repetitions.

LR models were trained on standardized predictors with the following settings: fit_intercept = True. We also trained gradient boosting regressor (GBR) and XGB using standardized inputs for consistency across all the models, even though tree‐based approaches do not need feature scaling. We used RandomizedSearchCV with 5‐fold internal CV (n_iter = 200, scoring = “r2”) to find the best hyperparameters for GBR on the training data. The best parameters for the GBR were found to be learning_rate = 0.05, *n*_estimators = 16, and subsample = 0.6. This combination was used again during the hold‐out assessment to reduce computational costs and time and to ensure consistent model configurations across resampling iterations. The XGB model’s best hyperparameters, identified using the same RandomizedSearchCV, were max_depth = 6, n_estimators = 30, and learning_rate = 0.01, which were used for the final model. While efficient, this approach may introduce some bias toward the initial split.

With the LSTM model, the predictor and target variables were scaled prior to training, and the same scaling was applied to the test data, with an inverse transformation performed before evaluation. The LSTM model includes one LSTM layer (16 units) with dropout (0.2), a dense branch for static features (16 units), and a final regression layer. The model was trained using parameters such as the Adam optimizer (learning rate = 0.001), MSE loss, batch size = 8, and for up to epochs = 400. Early stopping and ReduceLROnPlateau were also used to lower the learning rate when the model was not showing performance improvement. There was no systematic adjustment of the LSTM model’s hyperparameters, and its settings stayed the same during the evaluation.

### 3.2. Overall Preclinical Performance Prediction

#### 3.2.1. Feature Contribution Analysis

**Figure 1:**
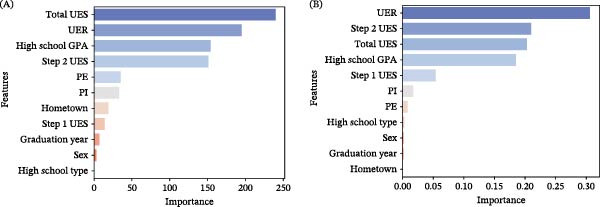
Feature importance analysis to measure the relative contribution of each feature to the preclinical score prediction. (A) SelectKBest with f_regression. (B) Random forest regression features important measures.

Figure 1 shows the relative contribution of demographic features and initial university scores for the admission to the prediction of average preclinical score over the years. It appears that the scores or rankings obtained have contributed more with respect to other demographic features, such as HSType, gender, graduation year, and hometown. Feature ranking and intrinsic feature importance score assessment by SelectKBest with f_regression and RF feature importance measure, respectively, confirm the low contribution of gender, graduation year, HSType, and hometown features. Because the admission indicators, such as Step 1 UES, Step 2 UES, total UES, and UER, are derived from related examination elements, correlation and multicollinearity diagnostics were examined prior to the interpretation of feature importance results. Therefore, the reported importance values should be interpreted as indicators of predictive contribution rather than independent causal effects of individual variables.

#### 3.2.2. ML Prediction Model

**Figure 2:**
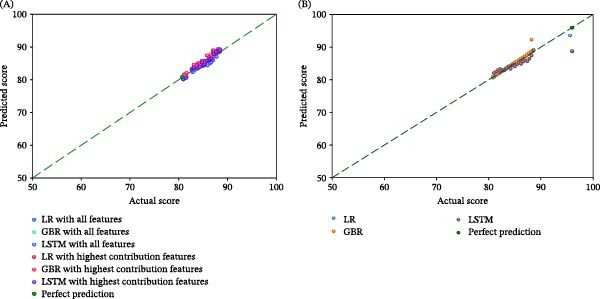
Prediction results for the (A) LR and GBR models, and (B) LSTM models, are shown using all the features as well as features with the highest contribution to the target variable were taken as the input variables. The green dashed line indicates the best‐fit line.

Overall performance prediction used the features shown in Figure 1, and various ML regression models were primarily tested to confirm the efficiency of the models in accurate prediction. Figure 2 shows the actual versus prediction results obtained from the LR, LSTM, and GBR regression models implemented in this study. The performance metrics recorded for the model’s prediction are reported in Table 2, which confirms that the models have low prediction errors and are performing well.

**Table 2:** Performance measures for the student performance prediction models.

Model	*R* ^2^ score	MAE	MSE
Case 1: All features reported are considered as input variables			
LR	0.81 ± 0.202	0.72 ± 0.170	0.73 ± 0.371
LSTM	0.82 ± 0.123	0.66 ± 0.223	0.70 ± 0.317
GBR	0.74 ± 0.181	0.80 ± 0.170	0.98 ± 0.372
Case 2: Features with the highest ranking are considered as input variables			
LR	0.82 ± 0.067	0.68 ± 0.082	0.69 ± 0.226
LSTM	0.82 ± 0.060	0.66 ± 0.071	0.70 ± 0.114
GBR	0.75 ± 0.135	0.80 ± 0.169	0.97 ± 0.454

#### 3.2.3. Year‐Wise Preclinical Student Performance

Here, the student performance prediction model utilized the bidirectional LSTM model, ensuring a balance between learning temporal dependencies and avoiding overfitting with dropout layers for regularization and dense layers for final predictions. Along with this, including callback classes such as ReduceLROnPlateau and EarlyStopping helped optimize training stability and prevent validation loss.

**Figure 3:**
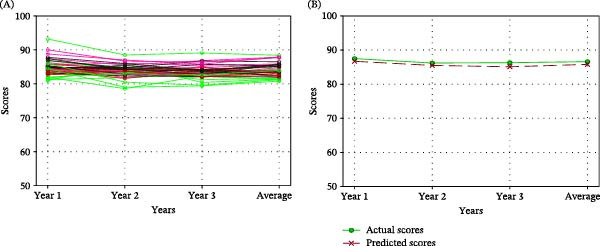
Trajectory plots with performance trend visualization of LSTM models for the test dataset. (A) All the features used as input variables for the models. (B) Trajectory of a single student over the course of the preclinical study.

Compared to the models discussed in section 3.1.2, we have considered preclinical scores of years 1–3 and average score as the target variables, while demographics and admission requirements are considered as input variables. Figure 3A indicates the year‐wise prediction outcomes when features with the highest contribution to the target variable were taken as the input variables, and Figure 3B visualizes the performance trend of a single student over the years of preclinical studies. Here, we can see an improvement in prediction accuracy over the years from year 1 to year 3 and in the prediction of the average score for the preclinical assessment. The outcome confirms how well the LSTM model generalizes across test data or unseen data and how various demographic features and admission criteria influence long‐term academic performance.

**Figure 4:**
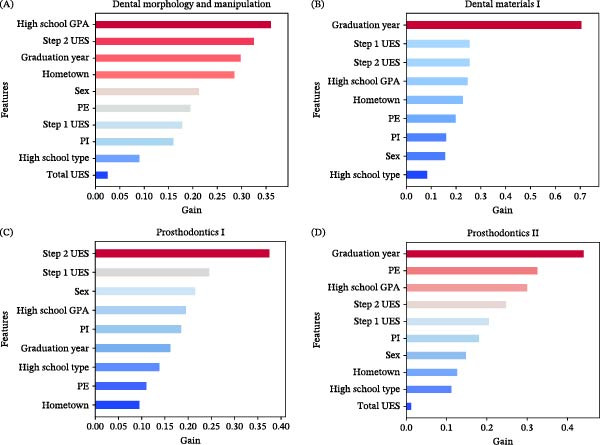
Feature importance analysis to measure the relative contribution of each feature to the preclinical subject’s performance prediction using XGB feature importance analysis. In the charts, gain indicates the relative contribution of each feature in generating the prediction. (A) Dental morphology and manipulation, (B) dental materials I, (C) prosthodontics I, and (D) prosthodontics II.

Across models, predictive performance was consistently higher for the aggregate 3‐year outcome (P_T) than for the single‐year Year 3 outcome (P_Y3), reflecting greater stability in averaged scores compared with year‐specific variability. Using five‐fold CV, LR and ensemble models achieved moderate‐to‐strong performance for P_T, whereas performance for P_Y3 was lower and exhibited greater variability across folds, indicating that Year 3 performance is harder to predict from admission/demographic features and prior‐year trends alone. The LSTM model, implemented with a valid temporal formulation of Year 1–2 scores as sequential inputs and static variables as auxiliary inputs, produced predictions comparable to the best‐performing tabular baselines for P_T. However, the predictions showed limited gains for P_Y3, which is consistent with the short sequence length and small sample size. Scatter plots for the predicted score versus actual score generated on the held out 20% test set also supported these findings, where the scatter plots were clustered around the identity line for P_T, while dispersion was larger for P_Y3. Trajectory plots for the test dataset and a representative single student demonstrated that the LSTM captures the overall direction of performance trends across years, although individual deviations remain, particularly for Year 3. Collectively, these results (Figures 3– 4) suggest that while admission and demographic variables can support useful estimation of overall preclinical performance, year‐wise prediction, especially Year 3, remains challenging and benefits only modestly from sequence modeling in this dataset.

### 3.3. Subject‐Wise Preclinical Performance Prediction

#### 3.3.1. Feature Contribution Analysis

Feature contribution in predicting the preclinical subject scores, such as for dental morphology manipulation, dental materials I, prosthodontics I, and prosthodontics II, was conducted using the plot_importance function of XGB models. As shown in Figure 4, this function helped to visualize how important each feature is in the trained XGB classification model [26]. Figure 4 indicates the consistent contribution of high school GPA, graduation year, Step 1 and Step 2 UES, in predicting the scores for individual preclinical subjects. Given the potential collinearity among admission variables, these feature importance measures should be interpreted cautiously as indicators of predictive contribution within the ML model rather than an independent explanatory influence.

#### 3.3.2. ML Prediction Models

The dataset is imbalanced because the reported letter grades (AA, BA, BB, CB, CC, and FF) do not have the same amount of information corresponding to the preclinical subjects (target variable). Considering the impact of imbalanced multiclass datasets in determining efficiency, the models were analyzed with precision, recall, and F1‐score, with the corresponding support, which is the number of actual instances of a class in the test dataset.

**Figure 5:**
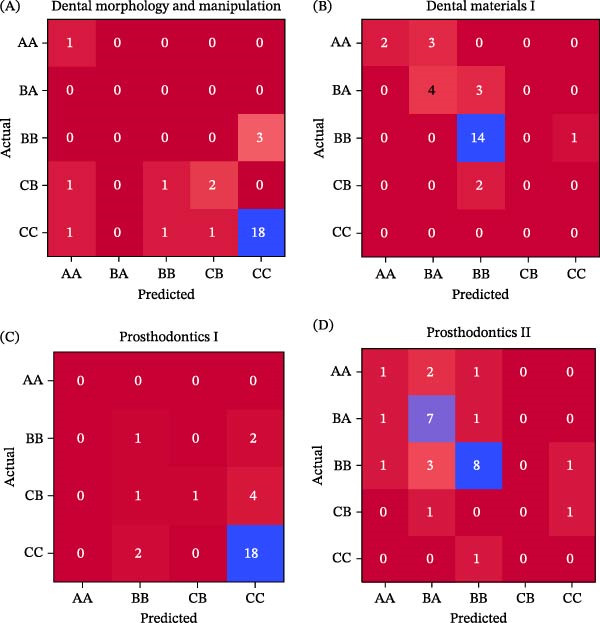
A confusion matrix that compares the actual class versus predicted classes by the multiclass classification model. Here, the rows correspond to the actual classes and columns correspond to the predicted classes. The confusion matrices for the subjects are (A) dental morphology and manipulation, (B) dental materials I, (C) prosthodontics I, and (D) prosthodontics II.

The following classification report (Table S1) and the confusion matrix (Figure 5A) are illustrations of the performance measures observed for each target variable with corresponding letter grade class outcomes. Results reveal that the models can perform the prediction of preclinical subject grades based on the demographic features and pre‐UESs. That means the preclinical grades are influenced by the students’ demographics and the performance on their pre university admission scores.

The XGB models’ results reported here are for the train:test data splitting ratio of 70:30. With the challenges of imbalanced class distribution, appropriate implementation of hyperparameter tuning and XGB model parameters such as regularization and early stopping helped to prevent overfitting issues to a great extent. For each target variable, considering the number of support data from each class (letter grades), the precision, recall, and F1‐score confirm the ML models’ capability in predicting the grades for preclinical subjects based on demographics and pre‐UESs. The F1‐score is an indicator of the harmonic means of precision and recall, which balances the trade‐off within an imbalanced set. High precision is an indicator of few false positives, and high recall is an indicator of few false negatives.

**Figure 6:**
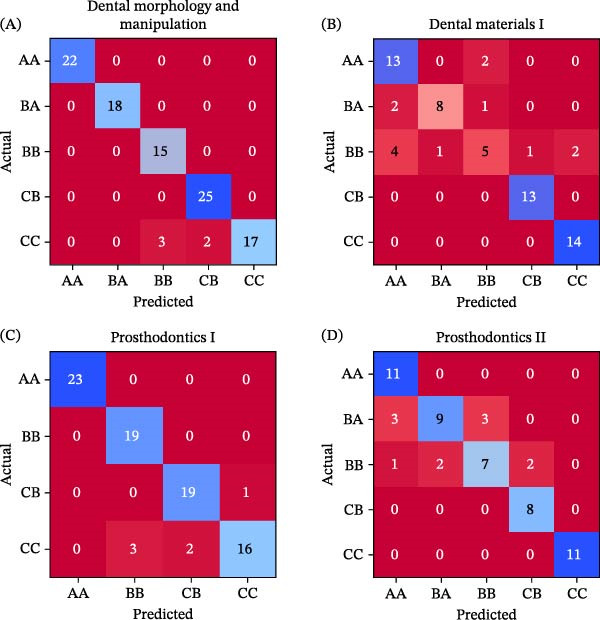
A confusion matrix that compares the actual class versus predicted classes by the multiclass classification model with a balanced dataset. Here, the rows correspond to the actual classes and columns correspond to the predicted classes. The confusion matrices for the subjects are (A) dental morphology and manipulation, (B) dental materials I, (C) prosthodontics I, and (D) prosthodontics II.

The results reported in Table S1 and Figure 5 show class imbalance problems. This issue is observed when we have classes with a rare amount of support compared to that of others. It is also observed that the accuracy score is better for classes that have a higher number of support data. An extension of this study is conducted to confirm the efficiency of the model across all the classes by implementing RandomOverSampler [27] resampling strategy to boost the impact of underrepresented or minority classes. Importantly, oversampling was applied only to the training dataset after the train–test split, while the test dataset retained the original class distribution to ensure unbiased evaluation. Table S2 and Figure 6 show the results from the corresponding implementation and confirm the capability of predicting the preclinical subject’s grades from demographics and pre‐UESs as the dependent variables, although these results should be interpreted as exploratory due to the limited dataset size (*n* = 96) and class sparsity. Due to the limited number of observations in some preclinical subject’s grade categories, several classes exhibited limited support and were occasionally absent from the test dataset. So, the classification results should be interpreted as exploratory findings demonstrating the feasibility of grade‐level prediction.

## 4. Discussion

In the present study, the effectiveness of different ML techniques in predicting the academic performance of preclinical dentistry students based on their pre‐university features was evaluated. The results of this study suggested that ML techniques may function as a supplementary tool for predicting the academic performance of dental students using their pre‐university records. Thus, the hypothesis of the present study that ML models would exhibit high accuracy in predicting preclinical dental students’ academic performances using their data was accepted.

Historically, admissions information has served as an indicator of academic success in dental school. Nonetheless, the admissions data can be analyzed to identify individuals who may be susceptible to inadequate academic achievement, and the determinants of success may differ from those that indicate failure [28]. Some studies have established a correlation between pre‐admission performance and subsequent academic success in dental school [20, 29–31].

Accurate predictive modeling can be achieved through various techniques, including regression, classification, and clustering. Nonetheless, it was stated that classification is among the most prevalent methods employed in forecasting academic success [4]. Previously, the prediction of academic success in pre‐clinical years of dentistry students was conducted utilizing three input parameters: age at enrollment, pre‐university cumulative GPA, and total semesters of matriculation. The ML models used in that study achieved accuracies ranging from 29% to 57%, ranked from highest to lowest as follows: RF, SVM, decision tree, and logistic regression. They also stated that pre‐university CGPA has been shown to predict the academic achievement of dentistry students; however, it alone did not yield optimal results [17].

The present study revealed that the UESs or rankings were more predictive than other demographic features, such as HSType, gender, graduation year, and hometown. ML techniques, including LR, LSTM, and GBR, achieved a relatively high *R* ^2^ score exceeding 70% for overall performance prediction and an accuracy beyond 70% for individual preclinical subject scores, depending upon the availability of a sufficient number of letter grades, as demonstrated by the comparison of Table S1 (imbalanced dataset) and Table S2 (balanced dataset) to support the training and testing phases. This result shows that the ML techniques used in our study are relatively more successful in predicting students’ academic performance.

This study has some limitations. First, the present study was conducted in a single institution in Turkey, and the dataset consisted of 96 observations only. The limited sample size restricts the development of universally acceptable predictive models due to the potential risk of overfitting, regardless of the use of CV and regularization strategies. Hence, the results of this study may not be generalized to other institutions or countries [17]. We also acknowledge that, in the classification models, several grade categories contain very small numbers of observations, which leads to limited support and occasional absence of certain classes in the test dataset. However, the investigation was designed as an exploratory feasibility study to determine if admission characteristics, demographic variables, and early academic performance trends yield sufficient indicators for students’ academic performance predictive modeling. Second, as the Sakarya University Faculty of Dentistry is a relatively newly established school, only the 2023 and 2024 grads’ data were examined. Future research with larger and more diverse datasets is necessary to broaden the scope of input factors and to provide a more thorough investigation of students’ academic achievement from different institutions across different countries, and thereby helps to validate the robustness and generalizability of these predictive relationships.

It is important to consider the fact that the inclusion of PI and PE requires explicit caution because these variables may act as proxies for socioeconomic advantage and may encode structural inequities. Their use may improve predictive accuracy, but it also raises fairness concerns, particularly if the model is framed as useful for admissions or other high‐stakes decisions. Accordingly, these variables should not be interpreted as merit‐based determinants of student potential. In this study, they were included for explanatory and predictive purposes only, and the model is not intended to support admissions decisions. If such models are used for early identification, they should be applied only to guide supportive interventions and should be accompanied by a fairness assessment and sensitivity analyses excluding socioeconomic variables.

Correlation analysis revealed moderate to strong relationships among many academic predictors, especially between UER, Step 1 UES, Step 2 UES, and total UES, with some correlations above |0.8|, indicating substantial multicollinearity. This is expected as these variables are all parts of academic success that overlap at different levels. This study did not use any formal methods for dealing with multicollinearity, such as eliminating variables or reducing dimensionality. Instead, all variables were kept for the following reasons. First, each variable captures a different part of academic achievement (such as individual exam scores versus cumulative indices), which was important for keeping the meaning clear and relevant to the field. Second, the primary models used in this study, tree‐based ensemble methods (gradient boosting and XGBoost) and DL (LSTM), are generally less sensitive to multicollinearity in terms of predictive performance as they do not rely on independent coefficient estimation like the linear models. Instead, they can accommodate correlated inputs through recursive feature splitting or nonlinear representations. However, it is acknowledged that multicollinearity can affect the feature importance, model stability, and particularly the interpretability of linear model coefficients. Therefore, results from the regression models need careful interpretation. Future work will explore diverse feature selection approaches, regularization, or dimensionality reduction techniques such as principal component analysis (PCA) to evaluate the impact of correlated predictors more comprehensively.

Investigating alternative ML methods and collecting bigger data may enhance the precision of predictions [18]. Incorporating these recommendations will improve prediction models in future studies and deepen the understanding of dental students’ academic performance.

## 5. Conclusion

ML technology may serve as a supplementary tool for predicting dentistry students’ performance. It can allow for the analysis and prediction of students’ academic performance through data mining prediction models. Using ML techniques in students’ academic performance prediction may provide insights into factors associated with academic performance and could potentially support educators to identify students in need of academic support and implement measures to improve academic performance and decrease failure rates within the dental education programs.

## Author Contributions

Conceptualization, formal analysis, investigation, methodology, project administration, resources, supervision, roles/writing – original draft, writing – review and editing: Remya Ampadi Ramachandran and Cortino Sukotjo. Conceptualization, formal analysis, investigation, methodology, roles/writing – original draft, writing – review and editing: Merve Koseoglu. Conceptualization, investigation, methodology, resources, roles/writing – original draft, writing – review and editing: Merve Botsali.

## Funding

No funding was received for this manuscript.

## Disclosure

All authors have read and agreed to the published version of the manuscript. The authors take full responsibility for the content of the manuscript.

## Ethics Statement

The study protocol was approved by the Sakarya University Faculty of Medicine research ethics committee (Protocol Number E–43012747‐050.04‐428167–162) and conducted in accordance with the 1964 Declaration of Helsinki.

## Conflicts of Interest

The authors declare no conflicts of interest.

## Supporting Information

Additional supporting information can be found online in the Supporting Information section.

## Supporting information


**Supporting Information** Table 1: Classification report providing a detailed summary of the machine learning model’s performance (precision, recall, F1‐score, and support) in predicting preclinical subjects’ letter grades across different dental courses using the original dataset. Table 2: Classification report for the balanced dataset, detailing the optimized performance metrics of the model in predicting preclinical subjects’ letter grades across the evaluated dental courses.

## Data Availability

The data are available upon request from the authors.
