# Supplementary material for: Machine Learning Methods to Predict the Academic Performance of Pre‐Clinical Dental Students Based on Pre‐University Information
Source: Int J Dent. 2026 Jun 11;2026:5468404. doi: 10.1155/ijod/5468404 (PMC13255521; doi:10.1155/ijod/5468404)
Supplement: Supplementary file 1 — Supporting Information Table 1: Classification report providing a detailed summary of the machine learning model’s performance (precision, recall, F1‐score, and support) in predicting preclinical subjects’ letter grades across different dental courses using the original dataset. Table 2: Classification report for the balanced dataset, detailing the optimized performance metrics of the model in predicting preclinical subjects’ letter grades across the evaluated dental courses. [file IJOD-2026-5468404-s001.docx]

**Supplementary Materials Table 1.** Classification report – a detailed summary of the performance of the model in predicting the preclinical subjects’ letter grades.

| **Dental Morphology Manipulation** | | | | |
| --- | --- | --- | --- | --- |
| Class | Precision | Recall | F1-score | Support |
| AA | 0.33 | 1.00 | 0.50 | 1 |
| BA |  |  |  |  |
| BB | 0.00 | 0.00 | 0.00 | 3 |
| CB | 0.67 | 0.50 | 0.57 | 4 |
| CC | 0.86 | 0.86 | 0.86 | 21 |
|  |  |  |  |  |
| accuracy |  |  | 0.72 | 29 |
| macro avg | 0.46 | 0.59 | 0.48 | 29 |
| weighted avg | 0.72 | 0.72 | 0.72 | 29 |
|  | | | | |
| **Dental Materials I** | | | | |
| Class | Precision | Recall | F1-score | Support |
| AA | 1.00 | 0.40 | 0.57 | 5 |
| BA | 0.57 | 0.57 | 0.57 | 7 |
| BB | 0.74 | 0.93 | 0.82 | 15 |
| CB | 0.00 | 0.00 | 0.00 | 2 |
| CC | 0.00 | 0.00 | 0.00 | 0 |
|  |  |  |  |  |
| accuracy |  |  | 0.69 | 29 |
| macro avg | 0.46 | 0.38 | 0.39 | 29 |
| weighted avg | 0.69 | 0.69 | 0.66 | 29 |
|  | | | | |
| **Prosthodontics I** | | | | |
| Class | Precision | Recall | F1-score | Support |
| AA |  |  |  |  |
| BA |  |  |  |  |
| BB | 0.25 | 0.33 | 0.29 | 3 |
| CB | 1.00 | 0.17 | 0.29 | 6 |
| CC | 0.75 | 0.90 | 0.82 | 20 |
|  |  |  |  |  |
| accuracy |  |  | 0.69 | 29 |
| macro avg | 0.67 | 0.47 | 0.46 | 29 |
| weighted avg | 0.75 | 0.69 | 0.65 | 29 |
|  | | | | |
| **Prosthodontics II** | | | | |
| Class | Precision | Recall | F1-score | Support |
| AA | 0.33 | 0.25 | 0.29 | 4 |
| BA | 0.54 | 0.78 | 0.64 | 9 |
| BB | 0.73 | 0.62 | 0.67 | 13 |
| CB | 0.00 | 0.00 | 0.00 | 2 |
| CC | 0.00 | 0.00 | 0.00 | 1 |
|  |  |  |  |  |
| accuracy |  |  | 0.55 | 29 |
| macro avg | 0.32 | 0.33 | 0.32 | 29 |
| weighted avg | 0.54 | 0.55 | 0.54 | 29 |

**Supplementary Materials Table 2.** Classification report for balanced dataset – a detailed summary of the performance of the model in predicting the preclinical subjects’ letter grades.

| **Dental Morphology Manipulation** | | | | |
| --- | --- | --- | --- | --- |
| Class | Precision | Recall | F1-score | Support |
| AA | 0.73 | 1 | 0.85 | 11 |
| BA | 0.82 | 0.6 | 0.69 | 15 |
| BB | 0.7 | 0.58 | 0.64 | 12 |
| CB | 0.8 | 1 | 0.89 | 8 |
| CC | 1 | 1 | 1 | 11 |
|  |  |  |  |  |
| accuracy |  |  | 0.81 | 57 |
| macro avg | 0.81 | 0.84 | 0.81 | 57 |
| weighted avg | 0.81 | 0.81 | 0.8 | 57 |
|  | | | | |
| **Dental Materials I** | | | | |
| Class | Precision | Recall | F1-score | Support |
| AA | 0.68 | 0.87 | 0.76 | 15 |
| BA | 0.89 | 0.73 | 0.8 | 11 |
| BB | 0.62 | 0.38 | 0.48 | 13 |
| CB | 0.93 | 1 | 0.96 | 13 |
| CC | 0.88 | 1 | 0.93 | 14 |
|  |  |  |  |  |
| accuracy |  |  | 0.8 | 66 |
| macro avg | 0.8 | 0.8 | 0.79 | 66 |
| weighted avg | 0.8 | 0.8 | 0.79 | 66 |
|  | | | | |
| **Prosthodontics I** | | | | |
| Class | Precision | Recall | F1-score | Support |
| AA | 1 | 1 | 1 | 23 |
| BA |  |  |  |  |
| BB | 0.86 | 1 | 0.93 | 19 |
| CB | 0.9 | 0.95 | 0.93 | 20 |
| CC | 0.94 | 0.76 | 0.84 | 21 |
|  |  |  |  |  |
| accuracy |  |  | 0.93 | 83 |
| macro avg | 0.93 | 0.93 | 0.92 | 83 |
| weighted avg | 0.93 | 0.93 | 0.93 | 83 |
|  | | | | |
| **Prosthodontics II** | | | | |
| Class | Precision | Recall | F1-score | Support |
| AA | 0.73 | 1 | 0.85 | 11 |
| BA | 0.82 | 0.6 | 0.69 | 15 |
| BB | 0.7 | 0.58 | 0.64 | 12 |
| CB | 0.8 | 1 | 0.89 | 8 |
| CC | 1 | 1 | 1 | 11 |
|  |  |  |  |  |
| accuracy |  |  | 0.81 | 57 |
| macro avg | 0.81 | 0.84 | 0.81 | 57 |
| weighted avg | 0.81 | 0.81 | 0.8 | 57 |
